# Effect of Harmonicity on the Detection of a Signal in a Complex Masker and on Spatial Release from Masking

**DOI:** 10.1371/journal.pone.0026124

**Published:** 2011-10-18

**Authors:** Astrid Klinge, Rainer Beutelmann, Georg M. Klump

**Affiliations:** Animal Physiology and Behavior Group, Department of Biology and Environmental Sciences, Carl-von-Ossietzky University Oldenburg, Oldenburg, Germany; Stanford University School of Medicine, United States of America

## Abstract

The amount of masking of sounds from one source (signals) by sounds from a competing source (maskers) heavily depends on the sound characteristics of the masker and the signal and on their relative spatial location. Numerous studies investigated the ability to detect a signal in a speech or a noise masker or the effect of spatial separation of signal and masker on the amount of masking, but there is a lack of studies investigating the combined effects of many cues on the masking as is typical for natural listening situations. The current study using free-field listening systematically evaluates the combined effects of harmonicity and inharmonicity cues in multi-tone maskers and cues resulting from spatial separation of target signal and masker on the detection of a pure tone in a multi-tone or a noise masker. A linear binaural processing model was implemented to predict the masked thresholds in order to estimate whether the observed thresholds can be accounted for by energetic masking in the auditory periphery or whether other effects are involved. Thresholds were determined for combinations of two target frequencies (1 and 8 kHz), two spatial configurations (masker and target either co-located or spatially separated by 90 degrees azimuth), and five different masker types (four complex multi-tone stimuli, one noise masker). A spatial separation of target and masker resulted in a release from masking for all masker types. The amount of masking significantly depended on the masker type and frequency range. The various harmonic and inharmonic relations between target and masker or between components of the masker resulted in a complex pattern of increased or decreased masked thresholds in comparison to the predicted energetic masking. The results indicate that harmonicity cues affect the detectability of a tonal target in a complex masker.

## Introduction

The ability to detect and process a sound in an acoustically complex environment, for example, a speech sound produced by one talker in an assembly of many talkers, is essential for efficient communication and the perception of important signals. However, the perception is often compromised by interfering masking stimuli. The amount of masking depends on the spectral and temporal characteristics of masker and target signal (further referred to as target). In a noise masker, for example, the amount of masking depends on the amount of masker energy present in the auditory filter that contains the target signal (but see also [1]). Energetic masking of signals by interfering sounds is an important factor of masking and has been widely studied [2]. However, other processes in addition to energetic masking mechanisms may affect the perception when detecting a target in a more complex masking stimulus than noise. Spatial cues such as time and intensity differences between the sounds reaching each ear can be exploited to considerably reduce the amount of masking if target and masker sound sources are at different spatial locations (an effect that is called spatial release from masking, SRM, e.g., [3], [4]). Furthermore, cues that result in perceptual grouping of target and masker components can affect masking considerably. Masking may be larger, for example, when target and masker are harmonically related (e.g., as typical for many musical instruments) or when target and masker share stimulus characteristics (e.g., speech in speech, [5]). On the other hand, masking may be reduced if target and masker belong to different harmonic tone complexes (e.g., a mistuned component in a harmonic masker, [6]). Finally, it has been observed that signal detection may be impaired in situations of high signal uncertainty, an effect that has been termed informational masking (e.g., [7], [8]).

While many psychophysical studies focused on energetic masking, informational masking or other masking effects related to binaural processing or auditory grouping alone, few studies have focused on the interaction of different masking effects [6], [9]. For example, Epp and Verhey [9] investigated the combined effects on the release from masking by comodulating the waveform envelope of different frequency bands (comodulation masking release, CMR) and by presenting signal and masker from various virtual locations (measurable as a binaural masking level difference, BMLD). Another study investigated the combined effects of harmonicity and stimulus uncertainty (i.e., the informational masking effect) on signal detection in the presence of a multi-tone complex masker [6].

In the natural listening environment in which the auditory system has to cope with overlapping sounds from multiple sources, various cues are simultaneously available and should enter in the analysis of the acoustic scene. Therefore, the current psychoacoustic study aims at investigating the combined effects of harmonicity of signal and masker components, their spatial origin and of stimulus uncertainty on the detection of signals in a complex multi-tone or noise masker in a free-field listening situation. The perceptual results are compared to estimated thresholds from a linear signal-to-noise ratio (SNR) model with a binaural component. By applying such a model we evaluate which amount of the observed masking can be explained by energetic masking and which other non-energetic masking effects have to be taken into account. To evaluate the role of harmonic relations between the signal and masker components on detection, we chose two harmonic and two inharmonic complexes that differed in their harmonic relation to the target and between components of the complex. A bandpass noise masker was applied as a non-harmonic control condition. To evaluate the influence of SRM on overall masking, we presented the pure tone target from the front or from the side while always presenting the masker from the front. To evaluate the influence of stimulus uncertainty, we compare detection thresholds of inharmonic maskers with a well predictable and an unpredictable spectral composition. Finally, the importance of the frequency range of signal and masker is evaluated since it will affect the monaural and binaural cues that can be exploited by mechanisms for spatial unmasking. The ability of the auditory system to extract interaural differences in the arrival time (ITDs) decreases with increasing frequency whereas interaural level differences (ILDs) become more important. The mechanisms available for detection of a signal in a complex multi-tone masker may differ whether signal and masker are presented in a frequency region that allows resolving the frequencies being presented or not. By studying this complex interaction of multiple cues on signal detection in the free sound field we may achieve a better understanding of masking effects in real-world listening situations.

## Materials and Methods

### Ethics Statement

Written consent was obtained from each participant prior to the experiments. The experiments were approved by the local ethics committee of the University of Oldenburg.

### Subjects

Five listeners - one male, four females, including the first author - participated in the experiments. They were between 21 and 31 years old (average of 25.6 years) and had normal hearing between 250 Hz and 8000 Hz (within 15 dB HL). Two subjects were experimentally naïve. Three subjects had previously participated in other psychoacoustic experiments, and one of these subjects (the author) obtained prior experience with the stimuli in a pilot experiment preceding the present study. Two of the five subjects have been musically trained. Each listener received at least two hours of training on the initial stimulus set before data collection began using the same paradigm as in the actual experiment. The subjects took between 5 to 15 days to complete the experiments.

### Procedure

A Go/NoGo paradigm with a continuously repeating background stimulus was used to determine the masked threshold of the sinusoidal signal. The masker alone was presented every 1.3 s forming the continuously repeated background stimulus. Pushing a button on the keyboard started a trial and initiated a random waiting interval of between one and seven seconds. After the waiting interval the test stimulus was presented which could either be the masker plus the simultaneously presented pure tone target signal (Go-stimulus) or the masker alone (NoGo-stimulus). The subject indicated the detection of the signal in the masker by pressing a second button on the keyboard. For each correct response (“Hit”) a visual feedback was given and the subject pushed the first button again to start the next trial. If the subject did not detect the signal in the masker, the trial was counted as a “Miss” and the next trial was initiated automatically. Trials in which the test stimulus was the masker alone (“catch trials”) occurred with a probability of 30%. A response of the subject within the response time during a catch trial was counted as a “False Alarm”. “Hit” rates and “False Alarm” rates were used to calculate the sensitivity measure d′ (see Data analysis).

A session consisted of 11 blocks of ten trials each with the first ten trials serving as warm-ups. During the warm-up block at the beginning of each session only the most salient test stimuli with the largest target-to-masker ratio and three catch trials were presented. Within each of the ten remaining blocks seven different test trials and three catch trials were presented in a randomized order. Target levels (step size 3 dB) were chosen according to the method of constant stimuli [10]. The range from the lowest to the highest target level was adjusted before each session to provide both sub-threshold and supra-threshold stimuli.

### Apparatus and stimulus generation

The free-field experiment took place in the anechoic room of the University of Oldenburg which fulfills the requirements for free-field measurements down to a lower cut-off frequency of 50 Hz. Subjects were placed on a seat in the middle of the chamber with their head position fixed by a head-rest mounted on the backrest of the chair. Two loudspeakers (Canton XS Plus) were placed 1.5 m from the subject's head, one at 0 degrees azimuth and the other at 90 degrees to the right. They were adjusted in height for each individual to maintain an elevation of 0 degrees. For visual feedback, a 15′ flat-panel display was placed next to but slightly behind the plane of the front loudspeaker. Subjects controlled the experiment via a standard keyboard.

On each day of experimenting, the setup was calibrated using a sound level meter (2238 Mediator, Brüel & Kjær) with the microphone placed at the position of the subject's head and facing the loudspeaker that was to be adjusted.

Stimuli were generated using a mobile Linux workstation and an RME sound card (Hammerfall DSP Multiface II connected via PCMCIA card). The output of the sound card was passed through a stereo amplifier (Harman Kardon HK 6350R) and sent to the two loudspeakers.

The target stimulus was a pure tone of 1 or 8 kHz starting in sine phase, with a duration of 125 ms including 25 ms Hanning ramps at stimulus onset and offset. Target frequencies of 1 and of 8 kHz were chosen to cover two frequency regions in which different attributes of sounds may be important. In the low frequency 1-kHz region ITDs would be expected to dominate localization performance, the components of the masker would be resolved (in separate auditory filters) and this frequency range plays an important role for the perceived quality of speech or music. In the high frequency 8-kHz region ILDs are the main binaural localization cue, the components of the masker are unresolved (interacting within an auditory filter) and this frequency region contributes to the perceived quality of speech or music, but to a lesser extent (see [11]). The target was switched on simultaneously with the masker which had the same duration and on- and offset.

**Figure 1:**
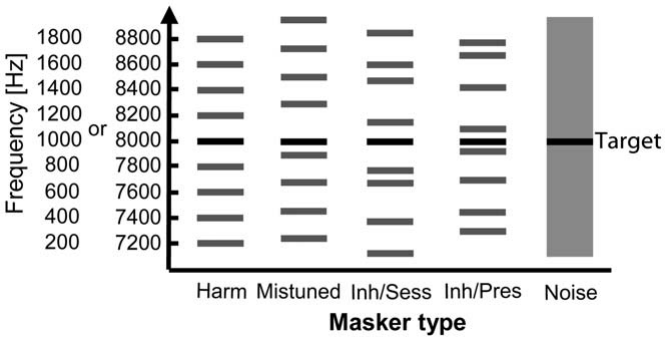
Schematic drawing of the five masker types used in the experiment. Schematic drawing of the five masker types (grey) in the two frequency regions used in the experiment as indicated on the ordinate. “Harm” = harmonic complex, “Mistuned” = mistuned compared to target, “Inh/Sess” = inharmonic, frequencies varied per session, “Inh/Pres” = inharmonic, frequencies varied per stimulus presentation, “Noise” = bandpass noise. See text for further explanations of the maskers. Target signals were 1 or 8 kHz pure tones, respectively (black).

We examined the effect of five different masker types on the detection of the target. Four of the five maskers were complex stimuli composed of pure tones starting in sine phase and the fifth masker was a bandpass noise masker (Fig. 1). The first masker type (“Harm”) was a harmonic complex with a fundamental frequency (F0) of 200 Hz comprised of four harmonics below and four harmonics above the target frequency (harmonic frequencies from 200 to 1800 Hz and from 7200 to 8800 Hz for the 1 and the 8 kHz target frequency, respectively). The second masker type (“Mistuned”) was a harmonic complex that also consisted of four harmonics below and four harmonics above the target frequency. The F0 for the target frequency of 1 kHz was 211 Hz (harmonic frequencies from 211 to 1899 Hz) and for the target frequency of 8 kHz it was 209 Hz (harmonic frequencies from 7106 to 8778 Hz). Due to the different F0 compared to the first masker type, the target tone was mistuned to the otherwise harmonic complex. The F0s for the low and high target frequency were slightly different to ensure a sufficient spacing between target frequency and adjacent harmonics of the masker. The third and fourth masker type (“Inh/Sess”: inharmonic per session, and “Inh/Pres”: inharmonic per presentation) were both maskers with inharmonic components that randomly deviated in frequency from the four harmonics below and above the target frequency presented in the harmonic complex masker. The difference between the two masker types was that for the “Inh/Sess” masker, a new frequency composition was used for each session that was conducted whereas for the “Inh/Pres” masker, a new frequency composition was used for every masker stimulus that was presented during a session (see Procedure). The frequencies for the “Inh/Sess” masker type were selected according to three conditions: (1) to be inharmonic to the target frequency, (2) to be in no harmonic relation to any other component at any F0, and (3) to lie within a pre-selected frequency of ±100 Hz around the nominal harmonic frequency. The frequencies for the “Inh/Pres” masker type were randomly selected within a frequency range of ±75 Hz around the nominal harmonic frequency to avoid frequency compositions with components too close to each other but without any of the other restrictions mentioned above. The frequency range for the “Inh/Sess” from which the frequencies for the components were drawn by a customized computer script had to be increased from originally ±75 Hz to ±100 Hz to provide enough variability in the selected frequencies. The sound pressure level of each harmonic component of the four complex masker stimuli was set to 60 dB SPL, which resulted in an overall masker level of 69 dB SPL. The fifth masker was a bandpass noise with a frequency range from 177 to 1910 Hz for the 1 kHz target frequency condition and from 6799 to 9287 Hz for the 8 kHz target frequency condition. The lower cutoff frequency was chosen to be at half an auditory filter bandwidth below the frequency of the first harmonic and the higher cutoff frequency was chosen to be at half a filter bandwidth above the frequency of the highest harmonic of the harmonic masker. The spectral density was set to 36 dB/Hz, which resulted in an overall noise masker level of 69 dB SPL. There was no spectral notch in the bandpass noise. At the beginning of each session a bandpass noise with a duration of 30 s was generated and for each stimulus presentation a 125-ms token was cut out at a random position to ensure that subjects would not learn a “frozen” noise token.

The maskers were always presented from the front loudspeaker. The pure tone target was either presented from the front (co-located configuration) or presented from 90 degrees from the right (spatially separated configuration). The resulting 20 conditions (2 target frequencies×2 spatial configurations×5 masker types) were divided into two experimental series – one for the 1 kHz target frequency and one for the 8 kHz target frequency.

### Data analysis

A session was accepted as being valid based on two conditions: (1) the two most salient stimuli must have an average hit rate of at least 80%, and (2) the false alarm rate must not exceed 20%. A psychometric function was constructed relating d′ to the level increment in the test trials. The threshold in a valid session was determined by linearly interpolating between adjacent points of the psychometric function as the level of the target resulting in a d′ of 1.8 [12]. Data from two consecutive valid sessions in which thresholds differed no more than 3 dB from each other were combined to determine the final masked threshold at a d′ of 1.8.

To ensure that training effects are excluded the presentation order of the experimental series (a series consisted of all conditions at one target frequency) as well as the order of the conditions within each series was randomized for each subject. Furthermore, after determining the last threshold in an experimental series the subject had to repeat the threshold measurement for the first condition of the series. If the threshold obtained for a second time differed by more than 3 dB from the threshold obtained the first time, then the next condition in series had to be repeated until the repeated threshold matched the threshold obtained the first time (difference ≤3 dB). Data were always taken from the threshold measurement of the repeated condition. The statistical software packages SPSS 17.0 and SigmaStat 2.03 were used to analyze the data.

### Model description

A simple, linear SNR model was used to predict the observed data. The model predicts a target threshold level at a given masker level assuming that the effective SNR within the auditory filter centered on the target is 0 dB at threshold.

In order to assess the effective SNR, the influence of spatial position on the input signals (pure tone target and the different maskers) was simulated using head-related transfer functions (HRTFs) measured from individual human subjects [13], [14]. The HRTF-filtered left and right ear signals for both target and masker were first passed through a bandpass filter, which coarsely simulates the transfer characteristics of the ear canal and the middle ear [15], and then through a gammatone filter [16] centered on the target tone frequency. The equivalent rectangular bandwidth (ERB) of the filter was calculated using the formula of Glasberg and Moore [17]: ERB = *f*/9.265+24.7 Hz, where *f* is the filter's center frequency in Hz. From the output of the gammatone filter, the SRM was calculated using the “equalization-cancellation” (EC) model by Durlach [18] in the analytical formulation [19]. The EC model estimates the SNR improvement that is possible by equalizing the interaural time and level differences of the masker and subsequently subtracting one ear signal from the other, thus reducing the masker level by destructive interference. The accuracy of the interaural time difference equalization is assumed to decrease towards higher frequencies due to the loss of phase locking. The spectral transfer function of the interaural cross-correlation low-pass in this study [19], which mimics the loss of phase locking towards high frequencies, was modified by introducing a constant maximal attenuation (a+1−a*exp(−σ∧2ω∧2)) at high frequencies instead of the continuously increasing attenuation (exp(−σ∧2ω∧2)) in the original model. The EC model provides the best SNR that is possible either by choosing the ear with the favorable SNR (“better ear listening”) or by binaural processing exploiting interaural time and level differences.

Thresholds were calculated for each experimental condition for each of the 51 individuals contained in the HRTF database. Thresholds for maskers with intrinsic statistical variation (i.e., for the noise and the “Inh/Pres” masker) were calculated five times and the resulting thresholds were averaged. Thresholds for all other conditions were calculated only once per individual of the HRTF database (with a new random frequency composition for the “Inh/Sess” masker for each subject).

## Results

### Effect of masker type on the masked thresholds

**Figure 2:**
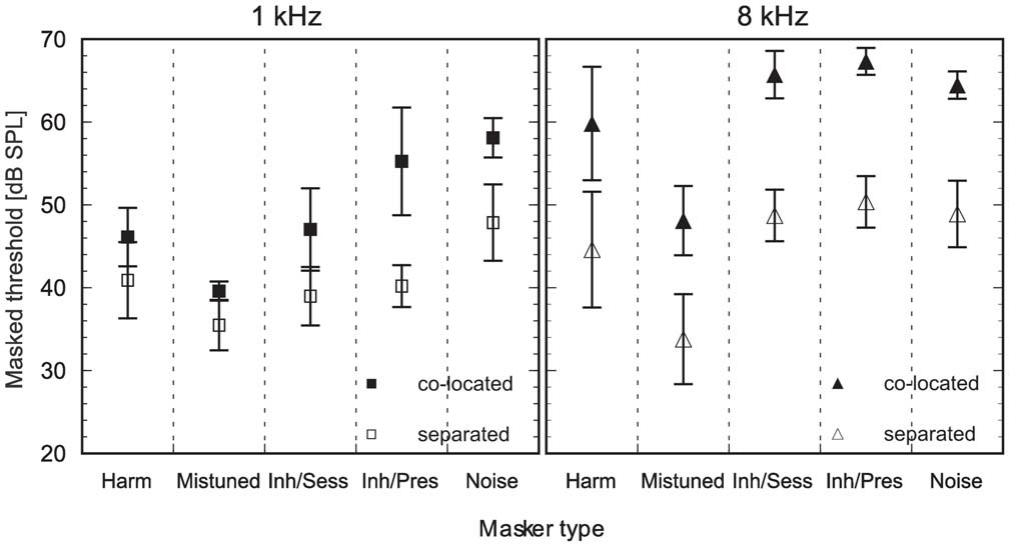
Mean observed masked thresholds for each masker type. Mean masked thresholds from five individuals for the detection of a pure tone target in each of the five masker types are displayed in absolute values (in dB SPL). Thresholds are shown for the co-located configuration (filled symbols) and for the spatially separated configuration (open symbols) separated by the target frequency (left panel = 1 kHz, right panel = 8 kHz). Error bars represent the standard deviation (SD). The five masker types used in this experiment are: “Harm” = a harmonic masker, “Mistuned” = a harmonic masker with an inharmonic relation to the target frequency, “Inh/Sess” = inharmonic per session, i.e. a random frequency composition redrawn for each session, “Inh/Pres” = inharmonic per presentation, i.e. a different random frequency composition for each stimulus presentation, “Noise” = bandpass noise.

The mean observed masked thresholds for each of the five masker types and each frequency region are shown in Figure 2. A repeated measures ANOVA revealed significant main effects of the within-subject factors target frequency, spatial configuration and masker type on the masked thresholds (target frequency: F_1,4_ = 22.71, p<0.01; spatial configuration: F_1,4_ = 101.00, p<0.01, masker type: F_4,16_ = 61.35, p<0.001). All two-way interactions were significant (p<0.05). An additional General Linear Mixed Model (GLMM) ANOVA that was applied to test for significant differences between individuals' performances and for effects of musical training revealed the same significant main effects as found with the repeated measures ANOVA but no significant effect of individual and musical training on the masked thresholds. Figure 2 shows that the masked thresholds differ for each masker type which is supported by the statistical analysis. The masked thresholds are significantly higher in the co-located configuration compared to the spatially separated configuration. Furthermore, the masked thresholds in the co-located configuration were always higher in the 8 kHz condition compared to the 1 kHz condition whereas the spatially separated configuration does not show this clear difference (Fig. 2). In Table 1 the observed thresholds are compared to the masked thresholds that were estimated using the SNR model described in Materials and Methods. The differences between the data predicted by the model simulations and the measured psychophysical results will be discussed with reference to additional literature data in detail in the Discussion.

**Table 1:** Comparison between observed and estimated masked thresholds.

Target frequency	Masker type	Estimated Masked Threshold		Observed Threshold		Estimated SRM	Observed SRM
		co-located	separated	co-located	separated		
**1 kHz**	Bandpass noise	59.6±1.3	50.3±1.6	58.1±2.4	47.9±4.6	9.3	10.2
	Harmonic	43.5±2.5	35.0±1.1	46.1±3.5	40.9±4.6	8.5	5.2
	Mistuned	48.3±2.4	39.3±1.1	39.6±1.2	35.5±3.0	9.0	4.1
	“Inh/Sess”	47.0±6.4	38.0±5.5	47.0±5.0	39.0±3.5	9.0	8.1
	“Inh/Pres”	44.1±3.4	35.3±1.8	55.3±6.5	40.2±2.5	8.8	15.1
**8 kHz**	Bandpass noise	63.8±1.6	55.7±6.8	64.5±1.7	48.9±4.0	8.1	15.5
	Harmonic	64.2±2.0	56.3±6.8	59.8±6.8	44.6±7.0	7.9	15.2
	Mistuned	64.0±2.0	56.1±6.8	48.1±4.2	33.8±5.4	7.9	14.3
	“Inh/Sess”	64.1±2.0	56.4±6.9	65.7±2.9	48.7±3.1	7.7	17.0
	“Inh/Pres”	64.2±2.0	56.3±6.8	67.3±1.6	50.4±3.1	7.9	16.9

Observed and estimated masked thresholds (in dB SPL) were compared for each masker type, for all target frequencies and spatial conditions. The first four columns show the estimated and the observed masked thresholds as mean absolute values (in dB SPL) ± standard deviation. The last two columns show the estimated and the observed amounts of spatial release from masking (SRM) in dB that were calculated by subtracting the thresholds of the spatially separated conditions from the thresholds of the co-located conditions. Mean values for the observed thresholds were calculated from five individuals. Mean values for the estimated thresholds were calculated from 51 subjects of the LISTEN HRTF database [13]. Details about the estimation of the thresholds can be found in the section Materials and methods: Model description.

Prior to further analyses the data were divided into four subgroups (1 kHz co-located, 1 kHz separated, 8 kHz co-located, and 8 kHz separated) as the main statistical analysis revealed a significant main effect of the target frequency and the spatial configuration on the masked thresholds and significant two-way interactions. In order to answer specific questions about the effect of harmonic or inharmonic structures of the masker on the masked thresholds, planned comparisons were performed (results of the statistical analysis in Table 2). This set of comparisons was constructed based on *a priori* hypotheses that will be explained for each contrast in the following paragraphs.

**Table 2:** Statistical results of the four planned contrasts for each of the four subgroups.

	Contrast 1	Contrast 2	Contrast 3	Contrast 4
subgroups	noise vs. complex	harmonic vs. inharmonic	“Harm” vs. “Mistuned”	“Inh/Sess” vs. “Inh/Pres”
**1 kHz co-located**	F = 129.6	F = 12.8	F = 17.9	F = 17.9
	p<0.001, η^2^ = .97	p<0.05, η^2^ = .76	p<0.05, η^2^ = .82	p<0.05, η^2^ = .82
**1 kHz separated**	F = 31.7	F = 2.5	F = 4.9	F = 2.2
	p<0.01, η^2^ = .89	p = 0.19, n.s.	p<0.05, η^2^ = .55	p = 0.211, n.s.
**8 kHz co-located**	F = 5.5	F = 51.8	F = 34.8	F = 2.2
	p = 0.079, n.s.	p<0.01, η^2^ = .93	p<0.01, η^2^ = .90	p = 0.208, n.s.
**8 kHz separated**	F = 4.3	F = 46.4	F = 29.6	F = 1.9
	p = 0.106, n.s.	p<0.01, η^2^ = .92	p<0.01, η^2^ = .88	p = 0.243, n.s.

A repeated measure ANOVA with planned contrasts was performed for each of the four subgroups and the F-value, the p-value and the effect size partial eta squared are displayed for each contrast. Details about the underlying hypotheses can be found in the results section.

At first we need to know if detecting a pure tone target in a complex tonal stimulus significantly differs from detecting a pure tone target in a noise masker. Thus, the bandpass noise masker was compared to the group of the four complex stimuli for the first planned contrast. At 8 kHz, the spectral energy in the filter was similar for the noise and the complex maskers. However, the bandpass noise masker lacked many of the cues present in the complex stimulus maskers: All complex stimulus maskers had distinct spectral peaks that were invariant throughout the presentation of each stimulus, and the “harmonic”, “mistuned”, and “Inh/Sess” maskers had an invariant temporal pattern over the course of a session which might influence the detectability of the pure tone in the masker. In addition, the components of the “harmonic” and the “mistuned” masker had a common fundamental, a feature that is not found in the noise masker. For the 1 kHz condition we would expect higher masked thresholds for the noise than for the complex maskers as the spectral energy in the filter centered at the target frequency in the 1 kHz condition was higher in the noise masker compared to the complex maskers. The planned contrasts revealed that the masked thresholds for the noise masker were significantly higher than those of the complex stimuli maskers in the 1 kHz condition but not in the 8 kHz condition.

The second planned contrast was constructed to tackle the question if a harmonic relation between the components of the masker influences the detection of the target in the masker. Thus, the four remaining complex maskers were divided into a harmonic complex stimulus group (“Harm” and “Mistuned” with components having a common fundamental) and an inharmonic complex stimulus group (“Inh/Sess” and “Inh/Pres”), and the masked thresholds of both groups were compared with each other. Without harmonicity as a grouping cue in the inharmonic masker types it was hypothesized that subjects perform worse compared to the harmonic complex maskers. The second contrast revealed significant differences between the two harmonic and the two inharmonic maskers in all but the subgroup “1 kHz spatially separated” (Table 2).

The third contrast (between the harmonic and the mistuned masker) was based on two hypotheses. First, the inharmonicity between the mistuned masker and the target should provide an additional cue (e.g., beating between components or a roughness in the timbre of the stimulus, [20]) and result in an increased detectability of the target in the co-located masker as well as in an improved segregation in a spatially separated configuration. Secondly, a harmonic relation between the harmonic complex masker and the target should have a strong grouping effect and therefore, should decrease the detectability of the target. Thresholds for the mistuned masker tended to be the lowest from all masker types for every condition and spatial configuration. The planned contrasts revealed that thresholds were significantly higher for the harmonic compared to the mistuned masker for all four subgroups (Table 2).

The fourth contrast was designed to compare the masked thresholds between the “Inharmonic per session” (“Inh/Sess”) and the “Inharmonic per presentation” (“Inh/Pres”) masker. If masker and target come from the same direction the high stimulus uncertainty in the “Inh/Pres” masker due to a perpetually varying frequency composition for every stimulus presentation should result in increased masked thresholds compared to the “Inh/Sess” masker in which the frequency composition only changed with every session. According to several studies that used highly uncertain masker types (informational maskers), the spatial separation of target and masker should provide a release from informational masking and thus an increased SRM at least in low-frequency regions can be expected (e.g., [7], [8], [21], [22]). However, the planned comparison only revealed significant differences between thresholds of both masker types for the subgroup “1 kHz co-located”.

### Effect of masker type on the spatial release from masking

**Figure 3:**
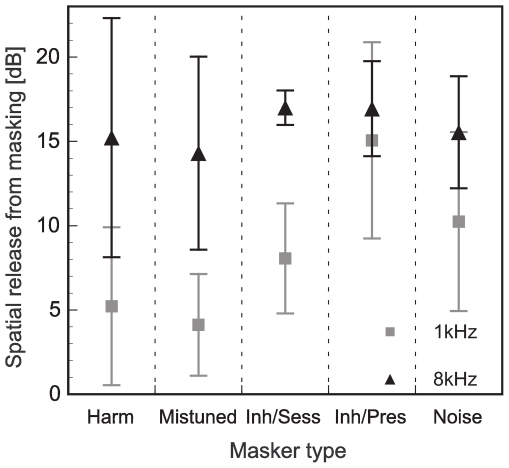
Amount of spatial release from masking for each masker type. Amount of spatial release from masking (difference between co-located and spatially separated configuration) for the 1 kHz target frequency (grey, squares) and for the 8 kHz target frequency (black, triangles) for each masker type. Error bars represent the standard deviation.

The SRM was calculated by subtracting the thresholds of the spatially separated configuration (open symbols, Fig. 2) from the thresholds of the co-located configuration (filled symbols, Fig. 2) for each masker type and at each target frequency. Figure 3 shows the mean SRM for the two target frequencies for each of the five maskers. In general, the SRM was always higher for the high-frequency condition (8 kHz target frequency, black triangles in Fig. 3) than for the low-frequency condition (1 kHz target frequency, grey squares in Fig. 3). A repeated measures ANOVA revealed significant main effects of the target frequency (F_1,4_ = 352.984, p<0.001) and the masker type (F_4,16_ = 3.27, p<0.05) on the amount of SRM, but there was no significant interaction (F_4,16_ = 2.79, p = 0.062). As the target frequency had a significant main effect on the amount of SRM, the data were divided into two subgroups (1 kHz and 8 kHz target frequency) for further statistical analysis. A repeated measures ANOVA with a Tukey's HSD *post hoc* test was performed within each of the two subgroups to evaluate which of the masker types differed significantly from each other in their amount of SRM. In the 1 kHz subgroup, the main effect of masker type was significant (F_4,16_ = 5.3, p<0.01). However, the only significant differences in the pair-wise comparisons were found for the “Inharmonic per presentation” masker compared to the harmonic masker (p<0.05) and to the mistuned masker (p<0.01). In the 8 kHz subgroup, the repeated measures ANOVA revealed no significant main effect of the masker type on the amount of SRM and no *post hoc* tests were made.

## Discussion

### Influence of masker type on the detection threshold

The current experiment showed that the detection of the sinusoidal target in the masker depended significantly on the type of the masker. However, the planned comparisons showed that not all thresholds of the different masker types were significantly different from each other (Table 2). As expected from the sound energy within the auditory filters at the target frequencies, the thresholds in the noise masker were significantly higher than the thresholds in the four tonal complex maskers in the 1 kHz conditions but not in the 8 kHz conditions. A comparison between the harmonic and the inharmonic masker types revealed a significant difference in all but the spatially separated 1 kHz condition. Within the harmonic maskers, the inharmonic relation between the harmonic masker and the target significantly decreased the masked threshold in all but the spatially separated 1 kHz condition. Within the inharmonic maskers, the only significant difference between the “Inh/Sess” and the “Inh/Pres” masker was found in the co-located 1 kHz condition.

By looking at the comparisons between the observed and the estimated masked thresholds (Table 1; for details about the model see Materials and methods) and at the results of the planned comparisons between the different groups of maskers (Table 2) a more complex pattern is revealed. We will discuss our findings while focusing on three main points. First, we want to investigate to which extent the observed amount of masking depends on a purely energetic masking effect as predicted by the model. Second, we are interested if the differences that are found between the masker types depend on their harmonic or inharmonic structure. Third, we want to examine if the frequency region influences the detection of the pure tone signal in the different masker types. In the following, the responses are discussed with reference to the masker types.

For the noise masker, the masked thresholds at the co-located configuration were well predicted by the model (Table 1). This result suggests that the target in a noise masker is primarily masked in relation to the spectral energy in the filter. Furthermore, the significantly higher masked thresholds in the noise compared to the complex maskers in the 1 kHz condition also supports the hypothesis that the masking depends primarily on energetic masking effects. In the 8 kHz conditions, there was no significant difference between the thresholds for the noise compared to the thresholds of the group of complex maskers indicating similar amounts of masking by both tonal complex stimulus and noise maskers. The distinct spectral peaks that were invariant throughout the presentation of each complex masker stimulus apparently did not help to improve the detection of the target in the complex masker.

For the harmonic masker (“Harm”), the harmonic relation between the components and between the masker and the target was hypothesized to be a strong cue that is able to group the target to the masker resulting in an increased masked threshold. The observed and the estimated thresholds deviated in different ways for the two target frequencies. In the 1 kHz condition, the observed masked threshold for the harmonic masker was about 3 dB higher than estimated for the co-located configuration. In the 8 kHz condition, however, the harmonic relation between all components seemed to have the opposite effect and the observed threshold was about 5 dB lower than predicted (Table 1). Thus, the assumption that the detectability of the target in the masker decreases due to the strong grouping provided by harmonicity is only supported in the lower frequency region. In the 8 kHz condition, the observed lower threshold suggests that an additional cue resulting in a release from masking could be exploited by the subjects. Such an additional cue provided in an auditory filter with more than two harmonics interacting in the filter might be the change in the otherwise constant envelope of the temporal waveform when adding the target to the harmonic masker during a test stimulus. The ability to detect such a change in the temporal waveform has been suggested before [23], [24].

As predicted, the masked thresholds for the mistuned masker yielded the lowest thresholds compared to the other maskers (Fig. 2). The observed thresholds were about 8 dB (at 1 kHz) and 16 dB (at 8 kHz) lower than predicted by the model. These substantial improvements in masked thresholds indicate that mistuning provided a strong cue helping to detect the mistuned target in the otherwise harmonic masker. This result is also supported by the significant difference in thresholds between the harmonic and the mistuned masker for all four subgroups (Table 2). A different effect of harmonicity on detecting a target, in this case a temporal gap, has been found in a study from Leung et al. [25]. Mistuning a component of a complex impaired the detection of a temporal gap in one of the components of the complex whereas in the current study it increased the detectability of a pure tone in the “mistuned” masker. They attributed the observed deterioration of performance to a shared attention between the complex and the second auditory object arising from the mistuning. A similar effect does not seem to apply in the present study. For the low frequency region in which the harmonics of the complex masker are resolved, the enhanced detectability may have been mediated by the segregation of the mistuned target from the harmonic complex (e.g., [26]). The perceptual segregation of a low-frequency mistuned component might rely on an across-channel comparison of periodicity information [27]. In the 8 kHz condition in which harmonics are unresolved and the pitch of the complex masker is weak, the predominant cue might have been the change of the envelope of the temporal waveform that results from adding the target to the masker. It has also been suggested that a gradually changing waveform due to the mistuning of the target in relation to the otherwise harmonic masker can be used as a cue to detect the target in the masker [23], [24].

In the inharmonic per session masker, the observed thresholds were similar to the estimated masked thresholds in the co-located 1 kHz and 8 kHz condition. Furthermore, the observed threshold of the “Inh/Sess” masker in the co-located 1 kHz condition was similar to the one found for the harmonic masker and significantly lower than the threshold determined with the “Inh/Pres” masker. Despite the larger frequency range over which the frequency randomization was realized for the “Inh/Sess” compared to the “Inh/Pres” masker, the random frequency composition seemed not to impair the detection of the target in the masker. The inharmonicity between all components of the “Inh/Sess” masker and the target eliminates grouping cues which, as we suggest, increase the threshold in the harmonic masker. On the other hand, the constant temporal waveform over the course of one session which only changed when the target was added to the masker rather might have aided the development of a template during the presentation of the masker-alone-stimulus in the 1 kHz condition. The masker-plus-target stimulus could then have been compared to a stored spectral or temporal template [28]. In the 8 kHz condition, the similarity between observed and predicted thresholds indicates that a template could not be learned or would not have improved the detectability of the pure tone target. It also makes it unlikely that a change in the waveform and/or the envelope of the stimulus was used as a cue for detecting the target in the “Inh/Sess” masker as we suggested for the harmonic masker.

The assumption that the perpetually varying random frequency composition in the “Inh/Pres” masker imposes a high stimulus uncertainty which leads to a high amount of masking and thus to increased thresholds was supported only in the co-located 1 kHz condition. In this condition, the observed masked threshold was about 10 dB higher than predicted by the model and the threshold between the “Inh/Sess” and the “Inh/Pres” masker was significantly different (Table 2). The proposed additional masking effect has been shown and discussed previously (e.g., [29]) and has been termed “informational” masking. Informational masking is mostly referred to as a central masking problem [30], in which, for example, the observed threshold elevation could be due to a simple statistical problem where the build-up of a template is impeded due to the variability of the frequency composition from one stimulus presentation to the other. For the 8 kHz co-located condition, the observed threshold was similar to the estimated threshold, suggesting that the perpetually varying frequency composition of the “Inh/Pres” masker has no additional masking effect in this frequency region. It is possible that the frequency variation of ±75 Hz around the nominal harmonic frequency was too small compared to the frequency resolution of the peripheral auditory system to induce a high stimulus uncertainty [8], [30].

The results of the harmonic, mistuned and the “Inh/Pres” masker for the co-located 1 kHz conditions can be compared to results from a study by Oh and Lutfi [6]. In their study applying monaural headphone stimulation they showed that the amount of masking depended on whether the 1 kHz pure tone signal was presented in a harmonic, a mistuned (i.e., the target frequency was shifted to 1047 Hz, thus, being mistuned in relation to the harmonic masker) or an inharmonic masker whose frequency composition varied with every presentation. Similar to the results in the present study, the highest masked thresholds were obtained for inharmonic per presentation maskers, a lower masking effect was found for harmonic and the least amount of masking was found for mistuned maskers.

### Influence of masker type on spatial release from masking

By determining the amount of SRM we wanted to examine the extent to which the processing of harmonicity cues interacts with the processing of spatial cues when detecting a pure tone target in different complex masker types. Figure 2 and Figure 3 show that all conditions yielded a SRM but only for the 1 kHz condition the amount of SRM significantly differed between the various masker types. The amount of SRM at 8 kHz was similar for all masker types suggesting that harmonicity cues did not influence spatial cues when detecting a pure tone target in a masker stimulus. As expected, the mean SRM was larger for the 8 kHz than for the 1 kHz conditions due to the head shadow effect that is larger at high frequencies.

The audibility of a target in a spatially separated masker is determined by two main spatial effects: (1) monaural effects such as the “better ear” effect in which the target-to-masker ratio (TMR) is increased in one of the ears when the target comes from a different spatial location than the masker [31], [32], and (2) binaural effects that result from interaural time or level differences (ITD and ILD). Binaural models (e.g., the EC model [18] or the lateralization model [33]) were traditionally used to estimate the amount of spatial or binaural release from masking. It has been pointed out previously that they fail to accurately predict spatial or binaural release from masking in situations in which masker and target are similar in their spectro-temporal characteristics and additional informational masking occurs [32]. Thus, deviations of the observed SRM from the results of the modified EC model in the present study may point towards additional effects of masking or masking releases such as increased spatial release from informational maskers or an influence of harmonicity on spatial cues.

The EC model that was used in the present experiment to estimate masked thresholds for the co-located and the spatially separated configuration was relatively accurate in predicting the amount of SRM for a pure tone target in a noise masker in the 1 kHz condition. Similar results compared to the results for the 1 kHz condition of the present study have been obtained in a free-field study [34] and a headphone experiment [35]. In the 8 kHz condition, the model predicted less SRM than observed for the noise masker. This deviation can be explained if we look at the HRTFs at this frequency region. The simulated masked thresholds in the spatially separated 8 kHz condition highly depend on the exact angle of the sound incidence at such a high frequency, especially for pure tones. Small deviations of the subject's head position from the 90 degrees azimuth result in a large increase of interaural level differences. Thus, despite the head-rest we used, slight movements or deviations from the fixed head position of the subject may have occurred that could have yielded some additional binaural advantage. Due to the model's structure we can assume that any deviation of the estimated from the observed threshold for the noise masker would result in the same deviations for all other masker types.

A noticeable deviation of the observed from the predicted SRM was found for the harmonic and the mistuned masker at the 1 kHz target frequency. The spatial separation of the target from the masker led to only about 5 dB SRM which is about 3 dB less than would have been expected from the results of the EC model (Table 1). It is consistent with the observation by McDonald and Alain [36] that the perceptual segregation of a component in a complex by differences in spatial cues is similar for harmonic and mistuned components of a tone complex. However, a closer look reveals differences between the two masker types. While for the harmonic masker the observed threshold for the spatially separated configuration was increased compared to the estimated threshold it was the co-located configuration for the mistuned masker in which the threshold was considerably decreased (by 8 dB) compared to the estimated threshold. Thus, for the harmonic masker, harmonicity as a grouping cue seemed to only affect the detection of the pure tone target if it was spatially separated from the harmonically related masker. For the mistuned masker, the spatial separation may have reduced the ability to exploit inharmonicity as a cue to detect the pure tone target in the masker. Still, the mistuning cue could be exploited in both spatial configurations for improving masked thresholds. The release from masking determined with stimuli providing both mistuning and spatial separation was as large as the sum of the masking releases obtained using stimuli with mistuning and spatial separation each (for 1 kHz 10.5 dB vs. 11.5 dB; for 8 kHz: 26 dB vs. 27 dB, see also Table 1). Similar to our study Du et al. [37] found an additive effect of a spectral feature of the sound, i.e., fundamental frequency (F0) and spatial separation when subjects were asked to correctly identify two simultaneously presented vowels differing in F0 or location or both. A summation of masking releases, in the current experiment due to mistuning and spatial separation, has also been observed for comodulation and dichotic listening conditions in other studies and a serial processing of these cues in the auditory system was proposed [9], [38].

The “Inh/Pres” masker in the 1 kHz condition was the only condition in which the SRM was significantly enlarged compared to that obtained with the harmonic masker or the mistuned masker. The observed threshold for the co-located configuration was 11 dB higher than predicted by the model whereas this difference was reduced to 5 dB for the separated configuration (Table 1). Segregating the target from the “Inh/Pres” masker with its high stimulus uncertainty yields an additional release from masking that cannot be accounted for by purely energetic masking and mechanisms of binaural release from masking that were traditionally used to explain SRM in noise maskers (e.g., [7], [32], [39]). It has been suggested that such a spatial separation increases the ability of subjects to focus on the desired location and thus reduces informational masking [32], [40], [41].

The SRMs predicted by binaural processes implemented in the EC-model were generally similar or smaller than the SRMs predicted purely on the TMR in the “better ear”. This suggests that a monaural mechanism determines the SRM for those masker types for which similar observed and predicted SRMs were found.
